# Performance Analysis of Silica Fluidized Bed Membrane Reactor for Hydrogen Production as a Green Process Using CFD Modelling

**DOI:** 10.3390/membranes15080248

**Published:** 2025-08-18

**Authors:** Maryam Barmaki, Elham Jalilnejad, Kamran Ghasemzadeh, Adolfo Iulianelli

**Affiliations:** 1 Chemical Engineering Department, Urmia University of Technology, Urmia 57155-419, Iran; mmb.barmaki1378@gmail.com (M.B.); e.jalilnejad@uut.ac.ir (E.J.); 2 Institute for Materials and Processes, School of Engineering, University of Edinburgh, Edinburgh EH8 9YL, UK; 3 Institute on Membrane Technology of the National Research Council (CNR-ITM), via P. Bucci 17C, Rende, 87036 Cosenza, Italy

**Keywords:** fluidized bed membrane reactor, hydrogen production, silica membrane, CFD analysis, methanol steam reforming

## Abstract

The main aim of this study deals with the potential evaluation of a fluidized bed membrane reactor (FBMR) for hydrogen production as a clean fuel carrier via methanol steam reforming reaction, comparing its performance with other reactors including packed bed membrane reactors (PBMR), fluidized bed reactors (FBR), and packed bed reactors (PBR). For this purpose, a two-dimensional, axisymmetric numerical model was developed using computational fluid dynamics (CFD) to simulate the reactor performances. Model accuracy was validated by comparing the simulation results for PBMR and PB with experimental data, showing an accurate agreement within them. The model was then employed to examine the effects of key operating parameters, including reaction temperature, pressure, steam-to-methanol molar ratio, and gas volumetric space velocity, on reactor performance in terms of methanol conversion, hydrogen yield, hydrogen recovery, and selectivity. At 573 K, 1 bar, a feed molar ratio of 3/1, and a space velocity of 9000 h^−1^, the PBMR reached the best results in terms of methanol conversion, hydrogen yield, hydrogen recovery, and hydrogen selectivity, such as 67.6%, 69.5%, 14.9%, and 97.1%, respectively. On the other hand, the FBMR demonstrated superior performance with respect to the latter reaching a methanol conversion of 98.3%, hydrogen yield of 95.8%, hydrogen recovery of 74.5%, and hydrogen selectivity of 97.4%. These findings indicate that the FBMR offers significantly better performance than the other reactor types studied in this work, making it a highly efficient method for hydrogen production through methanol steam reforming, and a promising pathway for clean energy generation.

## 1. Introduction

Hydrogen is essential for a sustainable energy future, serving as a clean energy carrier for transportation, power generation, and industrial processes. Its production is crucial due to its potential to reduce greenhouse gas emissions and significantly replace fossil fuels [1]. Hydrogen combustion produces only water, making it a key player in climate change mitigation strategies. Additionally, hydrogen can be generated from water, natural gas, derived of biomass, and renewable sources, enhancing energy security by diversifying supply sources. Its importance extends to industrial applications like ammonia synthesis and petroleum refining, and it plays a pivotal role in emerging technologies such as fuel cells for electric vehicles [2,3].

Methanol steam reforming (MSR) is a promising method for hydrogen production. It operates at lower temperatures (200–300 °C) compared to steam methane reforming (700–1000 °C), leading to reduced operational costs and improved energy efficiency [4]. Methanol’s high hydrogen-to-carbon ratio results in a higher hydrogen yield per unit of feedstock, making it more efficient. Additionally, MSR produces fewer CO_2_ emissions than methane case, and the CO_2_ can be captured and reused in methanol synthesis, minimizing environmental impact [5]. Methanol can be derived from renewable sources like biomass and renewable electricity via CO_2_ hydrogenation, further enhancing sustainability. As a liquid at ambient conditions, methanol is easier and safer to store and transport than gaseous hydrogen, and existing fuel infrastructure can be adapted for its distribution. Recent advancements in catalyst development have improved the efficiency and selectivity of MSR, with catalysts now operating at lower temperatures and lasting longer, making the process more economically viable [4,5]. Figure S1 presented in the Supplementary File illustrates the growing interest in hydrogen production through the methanol reforming process, as evidenced by the increasing number of publications indexed in the Scopus database. Among different technologies, membrane reactors (MRs) have emerged as a significant innovation in hydrogen production through methanol reforming, integrating the reaction and separation processes into a single unit to enhance efficiency and performance [6,7]. They combine the methanol reforming reaction with hydrogen separation, continuously removing hydrogen and shifting the equilibrium to increase hydrogen yield and purity [8,9,10]. Various membranes, including palladium-based [11] and ceramic [12], have been developed, with palladium-based membranes being particularly noted for their hydrogen permeability and selectivity. Optimizing operating conditions such as temperature, pressure, and feed composition reduces energy consumption and costs. However, recent studies [13,14,15,16,17,18] suggest that silica MRs could be a promising alternative to Pd-based MRs for hydrogen production during MSR. Silica MRs offer environmental benefits by reducing CO_2_ emissions and improving energy efficiency. Economically, compared to Pd-based MRs, they promise lower operational costs, making them suitable for large-scale hydrogen production.

Despite the advancements, most research has focused on PBMR evaluation [11,12,13,14,15,16] for hydrogen production during MSR. On the other hand, FBMRs could be a promising technology for efficient hydrogen production. Their enhanced heat and mass transfer capabilities and effective hydrogen separation can lead to higher hydrogen yields and more efficient processes. However, further research and development are needed to overcome technical challenges for their widespread application [19].

Due to the high cost of experimental studies, utilizing theoretical analysis methods such as computational fluid dynamics (CFD) can be a cost-effective approach to achieving research objectives [20,21]. CFD allows detailed simulations and analyses of complex fluid flow and reaction processes, making it a valuable tool for studying reactor designs and performance [22]. To the best of our knowledge, there is currently no comprehensive study that evaluates the performance of FBMRs compared to PBMRs for hydrogen production. This represents a significant gap in the literature [20,21,22,23,24,25], as understanding the relative advantages and limitations of these reactor configurations is crucial for optimizing hydrogen production processes.

Therefore, this study analyzes hydrogen production capabilities of FBMR compared to PBMR, both integrated with microporous silica membranes, during the MSR process. This investigation is conducted using the CFD method to simulate and analyze the reactor performance under various conditions. Specifically, the effects of different operating parameters on methanol conversion, hydrogen selectivity, and hydrogen yield have been examined. These parameters include reaction temperature, reaction pressure, gas hourly space velocity (GHSV), and feed molar ratio. By comparing these parameters in both FBMR and PBMR configurations, we aim to provide a thorough evaluation of their performance relative to each other and conventional reactors (CRs) as well. The insights gained from this study could guide the development of more efficient hydrogen production systems and help identify the optimal reactor configuration and operating conditions for MSR.

## 2. Methodology

### 2.1. Reactor Design and Assumptions

**Figure 1:**
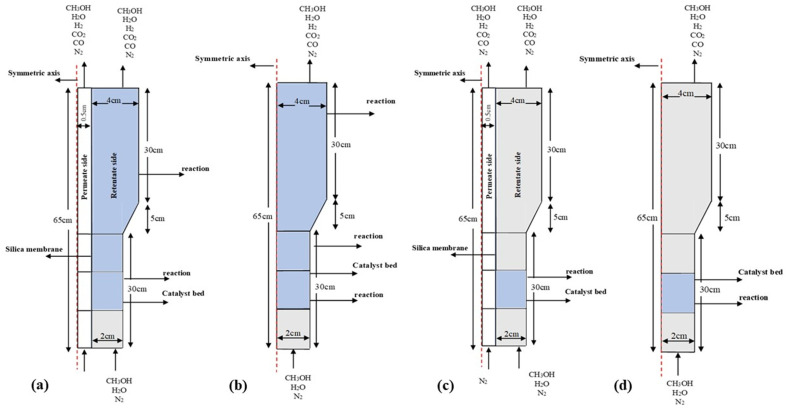
Schematic four reactor configurations simulated in this study; (**a**) Fluidized bed membrane reactor, (**b**) Fluidized bed reactor, (**c**) Packed bed membrane reactor, and (**d**) Packed bed reactor (all the dimensions are in cm).

Figure 1 presents the two-dimensional axisymmetric configurations of FBMR, PBMR, FBR, and PBR, which were considered in the modelling evaluation using the CFD method. For comparative analysis, the geometries of the MRs and CRs were kept identical. The MRs (Figure 1a,c) were modelled using a 2D domain with a height of 65 cm, a bottom module radius of 2 cm, a top module radius of 4 cm, and a silica membrane radius of 0.5 cm, using COMSOL Multiphysics 6.1. Similarly, for the CR configurations (Figure 1b,d), the only difference was the exclusion of the silica membrane within the reactor.

Main assumptions have been made to simplify the problem, while still capturing the essential physics as follows:

- Neglect 3D effects and focus on a 2D cross-section.
- Consider axisymmetric geometry.
- Incompressible flow (due to low Mach numbers).
- Uniform catalyst size distribution.
- Spherical shape for catalysts.
- Assumed to have a bubbling fluidization regime for the FBMR and FBR cases.
- Use empirical Schiller–Naumann drag model for the FBMR and FBR cases.
- Neglect radiative heat transfer.
- Simplified mass transfer model for silica membrane permeation (activated molecular sieve mechanism based on single gases permeation).
- Uniform catalyst distribution.
- No-slip condition at walls.
- Simplified inlet and outlet conditions.
- Use Darcy’s law or Forchheimer equation for porous media flow (in PBMR case).
- Constant overall bed porosity (in PBMR case).

It should be noted that the Schiller–Naumann drag model was employed in this study due to its established applicability to gas–solid systems and numerical stability within CFD code. Although more advanced models such as Gidaspow or EMMS may offer enhanced predictive accuracy, especially in catalytic fluidized beds, they were found to cause convergence challenges in the current setup. A comparative analysis of drag models and their impact on FBMR performance is planned as a future extension of this work.

Moreover, the permeation model used in this study adopts a simplified Arrhenius-type form with constant activation energy and pre-exponential factors, commonly used in membrane reactor CFD studies. Effects such as concentration polarization, competitive adsorption, and membrane fouling are not included, as the focus of this work is on comparative performance analysis. Incorporating such phenomena would require detailed experimental data and will be considered in future research.

These assumptions can help reduce computational complexity while maintaining the appropriate accuracy of FBMR and PBMR models.

### 2.2. CFD Modelling Approach

To develop an accurate CFD model, it is crucial to extract and solve the governing differential equations: continuity to track velocity changes, momentum balance to determine pressure along the reactor length, specious transport to monitor molar changes, and energy balance to calculate temperature variation.

#### 2.2.1. Governing Equations

- Continuity equation.

The continuity equation ensures mass conservation within the system:

(1) $$\frac{\partial \rho }{\partial t}+\nabla .(\rho .u)=0$$

where ρ is the fluid density, u is the velocity vector, and t is time.

- Momentum equation.

The momentum equation (Navier–Stokes equation) accounts for the conservation of momentum:

(2) $$\frac{\partial (\rho u)}{\partial t}+\nabla .(\rho .u.u)=-\nabla P+\nabla .\tau +\rho g+F_{d}$$

where P is the pressure, τ is the stress tensor, g is the gravitational acceleration vector, and F represents external forces such as drag forces, which are valid for fluidized bed configurations.

Indeed, in fluidized beds, interactions between gas and solid phases are significant. This includes the drag force model namely, the Schiller–Naumann model:

(3) $$F_{d,i}=\frac{3}{4}\frac{C_{D}\rho_{g}}{d_{p}}\left|u_{g}-u_{p}\right|(u_{g}-u_{p})$$

(4) $$C_{d}=\frac{24}{Re}(1+0.15{Re}^{0.687})$$

where F_d,i_ is the drag force on particle i, C_D_ is the drag coefficient, $\rho_{g}$ is the gas density, d_p_ is the catalysts particle average diameter, u_g_ is the continues phase velocity, u_p_ is the disperse phase velocity, and Re is dimensionless Reynolds number.

- Specious transport equation.

For multi-component systems, the specious transport equation describes the conservation of individual chemical species:

(5) $$\frac{\partial (\rho y_{i})}{\partial t}+\nabla .(\rho .u.y_{i})=-\nabla .J_{i}+R_{i}+S_{i}$$

where y_i_ is the mass fraction of species I, J_i_ is the diffusion flux of species i, R_i_ is the net rate of species i production due to chemical reaction, and S_i_ is the source/sink term of species i which are transferred by the silica membrane.

- Reaction rate correlations.

The reaction rate expressions and parameters have been taken from our previous study [14] and defined for the adjusted reaction zones in four reactor configurations (see Figure 1).

MSR reaction: (${CH}_{3}OH+H_{2}O↔{3H}_{2}+{CO}_{2}$)

(6) $$r_{\mathrm{SR}}=\frac{k_{\mathrm{SR}}k_{{\mathrm{CH}}_{3}O^{\left(1\right)}}^{*}(p_{{\mathrm{CH}}_{3}\mathrm{OH}}/p_{H_{2}}^{1/2})(1-\left(p_{H_{2}}^{3}p_{CO_{2}}/k_{\mathrm{SR}}^{\mathrm{eq}}p_{CH_{3}\mathrm{OH}}p_{H_{2}O}\right))C_{S1}^{T}C_{S1a}^{T}}{(1+k_{{\mathrm{CH}}_{3}O^{\left(1\right)}}^{*}(p_{{\mathrm{CH}}_{3}\mathrm{OH}}/p_{H_{2}}^{1/2})+k_{\mathrm{HCO}O^{\left(1\right)}}^{*}p_{{\mathrm{CO}}_{2}}p_{H_{2}}^{1/2}+k_{{OH}^{\left(1\right)}}^{*}(p_{H_{2}O}/p_{H_{2}}^{1/2}))(1+k_{H^{1a}}^{1/2}p_{H_{2}}^{1/2})}$$

Methanol decomposition (MD) reaction: (${CH}_{3}OH↔{2H}_{2}+CO$)

(7) $$r_{MD}=\frac{k_{\mathrm{MD}}k_{{\mathrm{CH}}_{3}O^{\left(2\right)}}^{*}(p_{{\mathrm{CH}}_{3}\mathrm{OH}}/p_{H_{2}}^{1/2})(1-\left(p_{H_{2}}^{2}p_{CO}/k_{\mathrm{MD}}^{\mathrm{eq}}p_{CH_{3}\mathrm{OH}}\right))C_{S2}^{T}C_{S2a}^{T}}{(1+k_{{\mathrm{CH}}_{3}O^{\left(2\right)}}^{*}(p_{{\mathrm{CH}}_{3}\mathrm{OH}}/p_{H_{2}}^{1/2})+k_{{OH}^{\left(2\right)}}^{*}(p_{H_{2}O}/p_{H_{2}}^{1/2}))(1+k_{H^{2a}}^{1/2}p_{H_{2}}^{1/2})}$$

Water gas shift (WGS) reaction: ($CO+H_{2}O↔H_{2}+{CO}_{2}$)

(8) $$r_{\mathrm{WGS}}=\frac{k_{\mathrm{WGS}}k_{{\mathrm{CH}}_{3}O^{\left(1\right)}}^{*}(p_{{\mathrm{CH}}_{3}\mathrm{OH}}/p_{H_{2}}^{1/2})(1-\left({p_{H_{2}}p}_{CO_{2}}/k_{\mathrm{WGS}}^{\mathrm{eq}}p_{CO}p_{H_{2}O}\right))C_{S1}^{T^{2}}}{(1+k_{{\mathrm{CH}}_{3}O^{\left(1\right)}}^{*}(p_{{\mathrm{CH}}_{3}\mathrm{OH}}/p_{H_{2}}^{1/2})+k_{\mathrm{HCO}O^{\left(1\right)}}^{*}p_{{\mathrm{CO}}_{2}}p_{H_{2}}^{1/2}+k_{{OH}^{\left(1\right)}}^{*}(p_{H_{2}O}/p_{H_{2}}^{1/2})}$$

The terms k_j_ and $\;K_{j}^{eq}$ represent the reaction rate and equilibrium constants for reaction j, respectively, while K_i_ denotes the adsorption coefficient for surface species i; $C_{S1}^{T}$ and $C_{S2}^{T}\;$ refer to the total catalyst surface concentrations for sites 1 and 2, respectively, and represent the total catalyst surface concentrations for modified sites 1a and 2a. All simulation data, reaction constants, thermodynamic data, and equilibrium constants are provided Tables S1 and S2 in the Supplementary File.

- Silica membrane permeation.

For silica membranes, a similar approach is applied using specific permeability values for each gas component. The flux of each component for silica membranes can be written as follows:

(9) $$J_{i}=Q_{silica,i}(P_{i,ret}-P_{i,perm})$$

where $P_{i,ret}$ and $P_{i,perm}$ are the partial pressure for species i in the retentate and permeate sides. $Q_{silica,i}$ can be calculated using the following equation:

(10) $$Q_{silica,i}\;=\;Q_{0,\;i}\exp(\frac{-E_{a,i}}{\mathrm{RT}})$$

where $Q_{0,\;i}$ is a pre-exponential coefficient and E_a,i_ the transport activation energy for each species; R and T represent gas constant and temperature, respectively. $Q_{0,\;i}$ and E_a,i_ for each species i are presented in Table 1.

#### 2.2.2. Boundary Conditions

In Table 2 and Table 3, the boundary conditions during simulations are presented.

#### 2.2.3. Post Processing Definitions

The following definitions have been used for describing and comparing the performance of each reactor:

(11) $$\mathrm{Methanol}\;\mathrm{conversion}\left(\%\right)=\frac{{CH}_{3}{OH}_{in}-{CH}_{3}{OH}_{out}}{{CH}_{3}{OH}_{in}}\cdot 100$$

where CH_3_OH_in_ and CH_3_OH_out_ are the methanol molar flow rate in the reactor inlet and outlet, respectively.

(12) $$\mathrm{Total}\;\mathrm{hydrogen}\;\mathrm{yield}\left(\%\right)=\frac{\left(H_{2-permeate}+H_{2-retentate}\right)}{3\cdot {CH}_{3}{OH}_{in}}\cdot 100$$

(13) $$\mathrm{Hydrogen}\;\mathrm{recovery}\left(\%\right)=\frac{H_{2-permeate}}{\left(H_{2-permeate}+H_{2-retentate}\right)}\cdot 100$$

(14) $$H_{2}-Selectivity\left(\%\right)=\frac{H_{2-permeate}+H_{2-retentate}}{3\times \left({CH}_{3}{OH}_{in}-{CH}_{3}{OH}_{out}\right)}.100\;$$

where H_2−permeate_ is the hydrogen molar flow rate that permeates through the membrane and H_2−retentate_ is the hydrogen molar flow rate in retentate side. It should be noticed that among the mentioned definitions, Equation (10) is related to the MRs simulation.

### 2.3. Solving Method and Mesh Independency

The governing equations of the CFD model were solved using the finite-element method, with pressure–velocity coupling corrections applied through the Semi-Implicit Method for Pressure Linked Equations (SIMPLE) algorithm. The computational model incorporates standard definitions for fluid and thermal properties, accounting for their dependencies on temperature, pressure, and composition. Specifically, all simulations were performed on a personal laptop equipped with an Intel^®^ Core^TM^ i7 10th generation processor, 32 GB RAM, and a 512 GB SATA SSD. The numerical solution was iterated until the tolerance value for all variables was reduced to less than 10^−4^. To ensure the independence of the simulation results from mesh size, preliminary tests were conducted under the following operating conditions: a reaction pressure of 1 bar, a reaction temperature of 573 K, an H_2_O/CH_3_OH ratio of 3, and a GHSV of 6000 h^−1^. These tests focused on methanol conversion across four different mesh densities, as detailed in Table S3 and Figure S2 via the Supplementary File. The mesh-independence analysis showed no significant differences in methanol conversion between the third and fourth mesh numbers for each reactor type. Therefore, the final mesh numbers selected for the simulations were 27,202, 16,077, 17,021, and 15,477 for the FBMR, PBMR, FBR, and PBR, respectively.

A structured quadrilateral mesh was used for all reactor models. No local refinement regions were employed, as the geometry and flow conditions were not expected to introduce highly localized gradients requiring additional mesh complexity.

### 2.4. Model Validation

**Figure 2:**
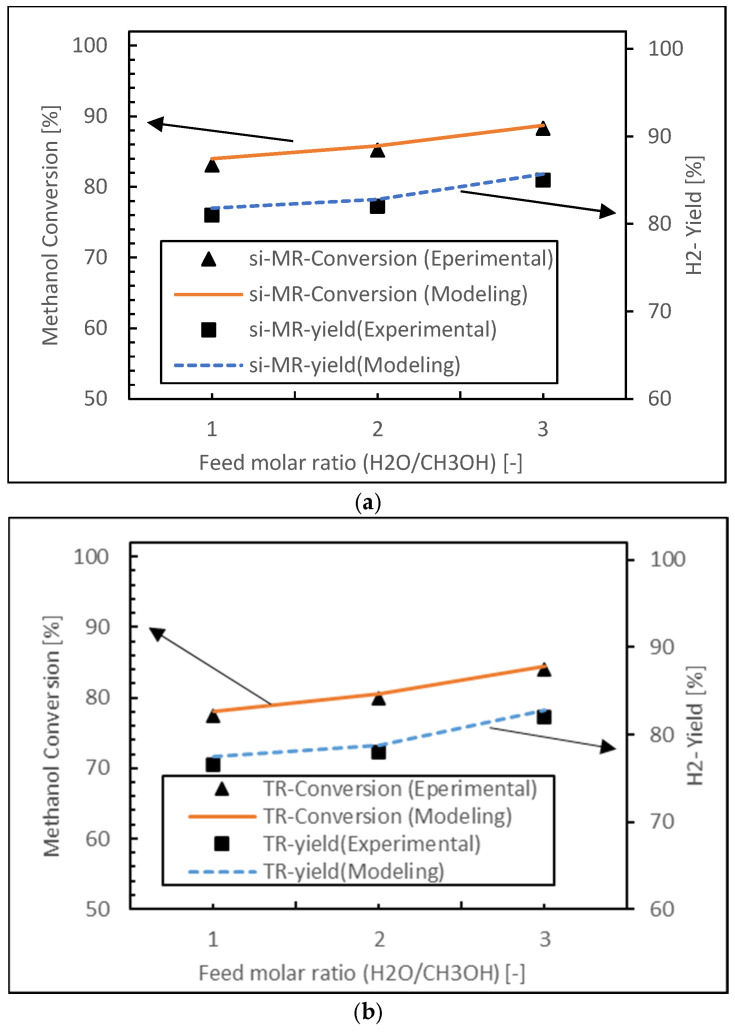
Comparison of methanol conversion in PBMR (**a**) and PBR (**b**) with experimental data at reaction pressure of 1 bar, reaction temperature of 573 K, GHSV of 6000 h^−1^, and H_2_O/CH_3_OH of 3.

In order to ensure the accuracy of the CFD model results, experimental validation was conducted. To achieve this, the theoretical predictions generated by the CFD model were compared with experimental data obtained in our previous study [13] (see Figure 2). This comparison was crucial in establishing the reliability of the model under the specific operating conditions. The systems and operating conditions in the CFD simulations of both PBMR and PBR operations were identical, specifically conducted at 573 K, with an H_2_O/CH_3_OH ratio of 3 and a GHSV of 6000 h^−1^.

The comparison results showed a minimum relative error of 1% and a maximum of 6.6%, both considered acceptable for the purposes of this study. This direct comparison between CFD predictions and experimental measurements validated the accuracy of the CFD model under these conditions.

While the CFD model was validated using available experimental data for PBR and PBMR configurations, direct validation of the FBMR model was not possible due to the lack of published experimental studies on methanol steam reforming in FBMR. This is a recognized limitation of the current study. However, the FBMR model was constructed using consistent modelling assumptions, governing equations, and kinetic/permeation parameters. The results should, therefore, be interpreted as theoretical predictions based on validated sub-models, offering comparative insights under equivalent conditions. Hence, future experimental work is essential to further validate and refine the FBMR model.

Even though the relative errors observed in model validation (ranging from 1% to 6.6%) indicate a strong agreement between simulation and experimental data, we acknowledge that a full uncertainty quantification and error propagation across the entire simulation domain and parametric variations were not conducted. This approach, although common in membrane reactor modelling studies, represents a limitation in our current work. The use of a deterministic modelling framework means that confidence intervals for hydrogen production or conversion metrics were not statistically derived. However, as the same modelling assumptions, boundary conditions, and numerical settings were applied uniformly across all compared configurations (e.g., packed vs. fluidized beds), the observed performance trends remain robust. For future work, incorporation of stochastic sensitivity analysis, Monte Carlo simulations, or global uncertainty propagation techniques could further enhance the confidence level in design optimization outcomes and provide probabilistic bounds for key performance indicators.

## 3. Results and Discussion

This study evaluates the effects of operating parameters—namely reaction temperature, reaction pressure, residence time, and feed molar ratio—on the performance of FBMR and PBMR in terms of methanol conversion, H_2_ yield, H_2_ recovery, and H_2_ selectivity. Subsequently, the flow regime of different MR configurations will be analyzed.

### 3.1. Effect Analysis of Operating Parameters

Based on the operating conditions outlined in Table 4, the performance of FBMR and PBMR will be investigated and compared with conventional FBR and PBR.

#### 3.1.1. Reaction Temperature Effect

Simulation studies were conducted to investigate the effect of reaction temperature on the performance of four reactors—fluidized bed, packed bed membrane, conventional fluidized bed, and conventional packed bed—in terms of methanol conversion, hydrogen yield, hydrogen recovery, and reaction selectivity for hydrogen. The reaction temperature was varied between 513 K and 573 K, while the reaction pressure was maintained at 1 bar, the feed molar ratio was set to 3, and the GHSV was fixed at 6000 h^−1^.

**Figure 3:**
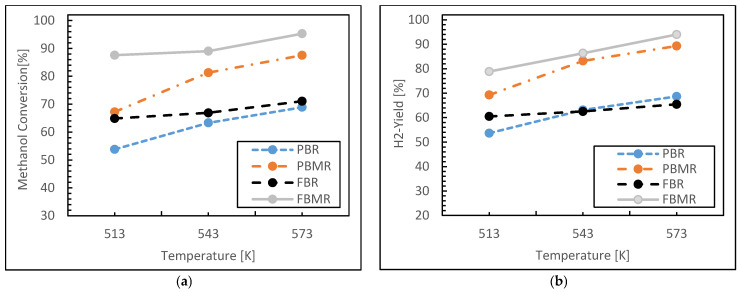
Methanol conversion (**a**) and Hydrogen yield (**b**) for FBMR in comparison with other reactors versus reaction temperature; at reaction pressure 1 bar, feed molar ratio is 3 and GHSV equals to 6000 h^−1^.

Figure 3a illustrates the methanol conversion percentages at various temperatures for the MSR process. Methanol conversion in all four reactors increases with temperature due to the endothermic nature of the reaction, as well as the corresponding increase in reaction rate at higher temperatures. MRs exhibit higher methanol conversion compared to conventional reactors, attributed to the continuous removal of hydrogen from the reaction zone through the membrane, which shifts the equilibrium towards greater product formation, consequently enhancing methanol consumption. However, at elevated temperatures, methanol and water vapor may also pass through the membrane, potentially lowering methanol conversion. Between 510 K and 540 K, the difference in performance between the reactors is minimal, while, above 540 K, the disparity becomes more pronounced.

For the PBR case, methanol conversion is approximately 50% at 510 K, increasing to around 65% at 570 K. The uniform heat transfer in PBR allows reactants to pass slowly through the catalyst bed, maximizing contact time and improving methanol conversion. According to what is stated above about the MRs behaviors, in the PBMR the selective permeation of products such as hydrogen enhances mass transfer and continuously shifts the reaction equilibrium towards product formation, thereby increasing methanol conversion.

In the FBR case, the continuous movement of catalyst particles ensures better mixing and contact with reactants, while superior heat transfer reduces temperature gradients. This results in fewer hot and cold spots, and leads to a higher methanol conversion rate. The FBMR shares these advantages, with the added benefit of reduced catalyst blocking, which is a common issue in packed bed reactors. The continuous motion of particles in the FBMR minimizes the risk of blockage, resulting in higher methanol conversion compared to other reactor configurations.

In addition, Figure 3b illustrates the relationship between hydrogen yield and reaction temperature, ranging from 513 K to 573 K. The FBMR and PBMR exhibit higher hydrogen yields compared to the conventional FBR and PBR across all temperatures. This aligns with the typical performance of MRs, where enhanced hydrogen separation and selective removal drive the reaction forward, particularly at elevated temperatures.

Hydrogen yield increases with temperature in all reactor types, indicating a positive correlation between temperature and hydrogen production. Among the reactors, the FBMR demonstrates the highest yield, achieving approximately 79% hydrogen yield at 573 K, while the conventional PBR shows the lowest yield, reaching only around 53% at the same temperature. The increase in temperature generally accelerates endothermic reactions, which contributes to higher hydrogen yields.

The superior performance of the FBMR can be attributed to the combination of fluidization, which enhances heat and mass transfer, and membrane separation, which shifts the reaction equilibrium toward greater hydrogen production. In contrast, the lower hydrogen yield of the conventional PBR may be due to the lack of these enhancements.

**Figure 4:**
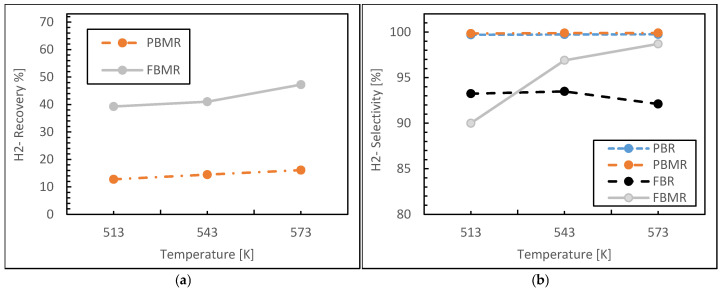
Hydrogen recovery (**a**) and Hydrogen selectivity (**b**) for FBMR in comparison with other reactors versus reaction temperature; at reaction pressure 1 bar, feed molar ratio is 3 and GHSV equals to 6000 h^−1^.

Figure 4a illustrates the variation of hydrogen recovery and reaction temperature in the FBMR and PBMR, as described by the hydrogen recovery concept (Equation (13)). According to the silica membrane flux equation (Equation (9)), an increase in temperature leads to higher hydrogen permeation, resulting in improved hydrogen recovery on the permeate side.

In the PBMR, hydrogen recovery is approximately 13% at 513 K, increasing to around 16% at 573 K, indicating that temperature has a relatively modest effect on hydrogen recovery in this reactor. In contrast, the FBMR demonstrates a more pronounced response to temperature. At 513 K, hydrogen recovery is about 40%, and it increases to approximately 47% at 573 K. This highlights that temperature has a more significant impact on hydrogen recovery in the FBMR compared to the PBMR. Although both MRs exhibit increased hydrogen recovery with rising temperature, the FBMR shows a substantially greater improvement. This can be attributed to enhanced hydrogen permeation, improved heat and mass transfer, and superior performance of the silica membrane at elevated temperatures.

Figure 4b presents the hydrogen selectivity as a function of reaction temperature for the four reactors. In the PBR, hydrogen selectivity increases with temperature between 513 and 573 K, consistently exceeding 99% across this range. This indicates that the conventional PBR configuration is highly effective for selective hydrogen production. Similarly, in the PBMR, hydrogen selectivity improves with temperature, reaching approximately 99.8% at 573 K. This high selectivity is expected, as MRs typically enhance selectivity by allowing the selective permeation of hydrogen, ensuring high purity.

In contrast, the conventional FBR shows a slight decrease in hydrogen selectivity, from around 93% at 513 K to approximately 92% at 573 K. This decline may be attributed to the occurrence of side reactions at higher temperatures or changes in reaction dynamics that reduce hydrogen selectivity. In the FBMR, hydrogen selectivity increases with temperature, rising from about 90% at 513 K to roughly 97% at 573 K. This improvement is likely due to the membrane’s enhanced separation efficiency at elevated temperatures, which helps to purify the hydrogen output.

In both the FBR and FBMR, the improved gas mixing and increased contact between reactants can lead to the formation of side products such as carbon monoxide and carbon dioxide, as observed in the component distribution during simulation. These by-products negatively impact hydrogen selectivity by reducing the proportion of hydrogen in the final product. Consequently, the hydrogen selectivity in these reactors is lower compared to the PBR and PBMR.

#### 3.1.2. Reaction Pressure Effect

The effect of reaction pressure on the performance of the four reactors—PBR, PBMR, FBR, and FBMR—was investigated in terms of object functions, which was already discussed. The reaction pressure was varied between 1 and 5 bar, while the reaction temperature was maintained at 573 K, with a feed molar ratio of 3 and a constant GHSV of 6000 h^−1^.

**Figure 5:**
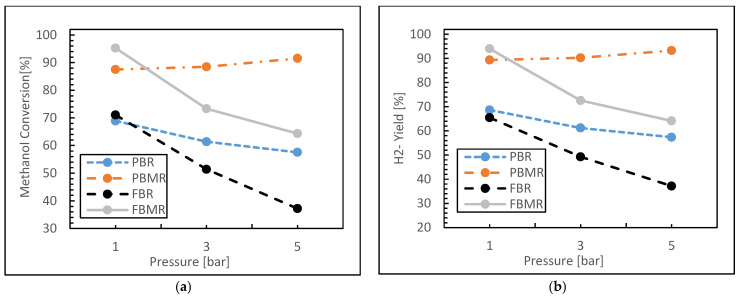
Methanol conversion (**a**) and Hydrogen yield (**b**) for FBMR in comparison with other reactors versus reaction pressure; at reaction temperature 573 K, feed molar ratio is 3 and GHSV equals 6000 h^−1^.

As shown in Figure 5a, methanol conversion for the MSR process decreases with increasing pressure in most reactor configurations. In the PBR, methanol conversion begins at approximately 68% at 1 bar and declines to around 57% at 5 bar, indicating a negative effect of increased pressure on conversion efficiency. This suggests that higher pressures are unfavorable for methanol conversion in the PBR.

In contrast, the PBMR maintains relatively high methanol conversion, starting at around 87% at 1 bar and increasing to approximately 91% at 5 bar. This high conversion is attributed to the membrane’s ability to effectively separate products, driving the reaction forward even at elevated pressures. For the FBR, methanol conversion shows a significant drop from around 71% at 1 bar to about 37% at 5 bar. This steep decline indicates that the FBR is highly sensitive to pressure increases, likely due to reduced contact between reactants and catalyst particles or re-mixing effects at higher pressures. In the FBMR, methanol conversion decreases steadily from about 95% at 1 bar to 64% at 5 bar. Despite the decline, the FBMR still exhibits relatively high conversion rates compared to the conventional PBR and FBR. This performance can be attributed to the membrane’s ability to selectively remove hydrogen, maintaining a low hydrogen partial pressure on the reaction side and shifting the equilibrium toward higher methanol conversion.

The selective removal of hydrogen through the membrane in both the PBMR and FBMR helps sustain high conversion rates even at elevated pressures. However, the FBMR is less effective than the PBMR in overcoming the thermodynamic limitations of the methanol reforming reaction at high pressures. Although both the PBMR and FBMR utilize membranes to selectively remove hydrogen and enhance methanol conversion, the fluidized nature of the FBMR introduces dynamic factors that reduce its ability to overcome the thermodynamic limitations at higher pressures. In contrast, the more controlled environment in the PBMR allows for more efficient hydrogen separation, enabling it to maintain higher methanol conversion rates even under elevated pressure conditions.

Figure 5b illustrates the hydrogen yield as a function of pressure for the four types of reactors discussed. In the conventional PBR, hydrogen yield decreases steadily from approximately 68% at 1 bar to about 57% at 5 bar. This decline suggests that increasing pressure adversely affects hydrogen yield in the conventional PBR, likely due to the shift in equilibrium toward the reactants in the hydrogen production reaction.

In contrast, the PBMR exhibits an increase in hydrogen yield with rising pressure, starting at about 89% at 1 bar and reaching 93% at 5 bar. This increase is attributed to the enhanced efficiency of the PBMR, which benefits from the membrane’s ability to displace the equilibrium toward hydrogen production, thereby improving hydrogen yield.

For the FBR, hydrogen yield decreases markedly from approximately 65% at 1 bar to around 37% at 5 bar. This significant reduction indicates that conventional FBRs are notably affected by pressure increases, potentially due to issues such as reduced contact effectiveness between reactants and catalysts at higher pressures.

In the FBMR, hydrogen yield starts at about 94% at 1 bar and decreases to approximately 64% at 5 bar. Despite this reduction, the FBMR maintains a relatively high hydrogen yield compared to both the conventional FBR and PBR. This superior performance is likely due to the membrane’s selective removal of hydrogen, which helps to partially counteract the negative effects of increasing pressure.

**Figure 6:**
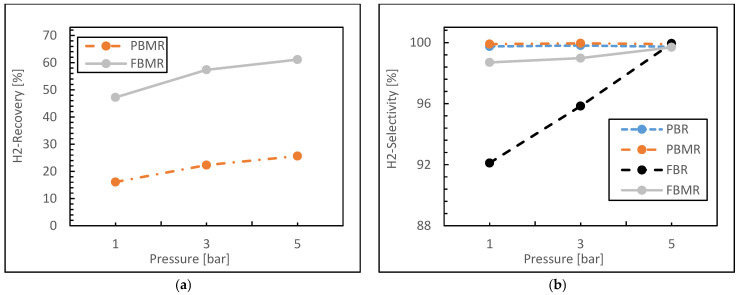
Hydrogen recovery (**a**) and Hydrogen selectivity (**b**) for FBMR in comparison with other reactors versus reaction pressure; at reaction temperature 573 K, feed molar ratio is 3 and GHSV equals 6000 h^−1^.

Figure 6a illustrates the variation in hydrogen recovery as a function of reaction pressure for two reactor configurations: FBMR and PBMR. In the PBMR, hydrogen recovery begins at approximately 16% at 1 bar and progressively increases to around 25% at 5 bar. This steady increase suggests a positive correlation between hydrogen recovery and reaction pressure. The increase in hydrogen recovery can be attributed to the enhanced selectivity of the silica membrane, which elevates the hydrogen separation factor and contributes to the driving force for hydrogen permeation. In contrast, the FBMR exhibits significantly higher hydrogen recovery, starting at around 47% at 1 bar and rising to approximately 61% at 5 bar. The more pronounced recovery with increasing pressure suggests that the fluidized bed configuration enhances both reactant–membrane interaction and the driving force for hydrogen permeation, primarily through increased flow turbulence. This, in turn, facilitates more efficient hydrogen recovery. Hence, these results demonstrate that the FBMR is more effective in hydrogen recovery compared to the PBMR under similar operating conditions.

Figure 6b presents the relationship between pressure and hydrogen selectivity during the MSR process for four different reactor types. In both the PBR and the PBMR, hydrogen selectivity remains consistently high, approaching nearly 99.8% across the entire range of pressure variations. The performance of these reactors is relatively stable, showing minimal sensitivity to pressure changes in these operating conditions. In contrast, the FBR exhibits a marked increase in hydrogen selectivity with increasing pressure, starting at approximately 92% at 1 bar and reaching close to 99.8% at 5 bar. This suggests that pressure has a more pronounced effect on hydrogen selectivity in the conventional FBR compared to the other reactor types. Similarly, the FBMR starts with high hydrogen selectivity, which gradually increases with pressure, following a trend similar to that of the PBMR, ultimately approaching 100%.

Additionally, simulation results confirm that both the PBR and PBMR demonstrate superior hydrogen productivity compared to other reactor configurations. The PBMR, in particular, achieves high hydrogen selectivity due to its use of membranes that selectively allow hydrogen permeation. Some studies [9] indicated that hydrogen selectivity in the PBMR could remain nearly constant with pressure during the reforming process at higher conversion values, as the membrane effectively removes hydrogen from the reaction zone, thereby preventing side reactions and by-products formation.

#### 3.1.3. Residence Time Effect

As another evaluation, the simulation results for the four reactor types—PBR, PBMR, FBR, and FBMR—are shown in terms of methanol conversion and hydrogen yield as a function of GHSV value.

**Figure 7:**
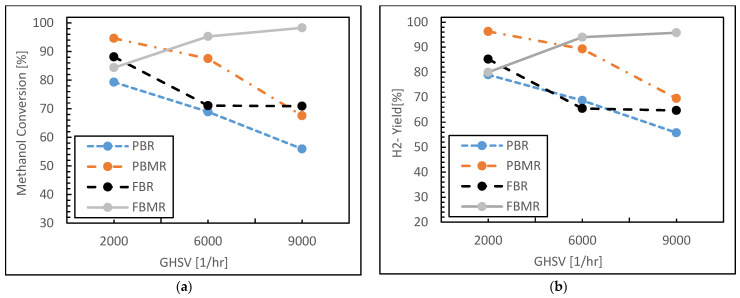
Methanol conversion (**a**) and Hydrogen yield (**b**) for FBMR in comparison with other reactors versus GHSV value; at reaction temperature 573 K, reaction pressure 1 bar, and feed molar ratio 3.

In the methanol conversion plot (see Figure 7a), the PBR shows a steady decline in performance as GHSV increases. Methanol conversion starts at around 75% for a GHSV of 2000 1 h^−1^ but drops below 60% at a GHSV of 9000 1 h^−1^. This decline suggests that at higher flow rates, the contact time between methanol and the catalyst is insufficient, leading to lower conversion rates. The PBMR, on the other hand, starts with a slightly higher methanol conversion rate of around 80%, and while it also declines as GHSV increases, it maintains a conversion rate of around 70% at higher GHSV values. This improved performance can be attributed to the selective removal of hydrogen by the membrane, which helps shift the reaction equilibrium and sustains higher conversion rates.

The FBR follows a similar declining trend but starts with a lower methanol conversion rate of around 70%. Its performance stabilizes slightly better than the PBR at higher GHSV values, remaining around 60%, likely due to better mixing and improved gas–solid contact in the fluidized bed, which enhances reaction kinetics. The FBMR, however, clearly demonstrates superior methanol conversion, starting near 90% and remaining above 80% even at higher GHSV values. The combined effects of the fluidized bed and the membrane-driven separation of hydrogen likely contribute to the FBMR’s consistently high performance, as both factors facilitate better mass transfer and drive the reaction equilibrium toward higher methanol conversion.

As depicted in Figure 7b, for the hydrogen yield concept, a similar pattern is observed. The PBR starts with a yield of around 75%, but as GHSV increases, the yield drops below 60%. The PBMR again outperforms the PBR, starting near 85% hydrogen yield and maintaining around 70% at higher GHSV values. The membrane in the PBMR plays a critical role in this higher yield, as it selectively removes hydrogen from the reaction zone, which prevents reverse reactions and side-product formation, sustaining higher hydrogen productivity.

The FBR shows a hydrogen yield comparable to its methanol conversion performance, starting at around 75% and decreasing with GHSV, but stabilizing above 60%. This result suggests that the fluidized bed’s improved gas–solid interaction enhances hydrogen production but cannot maintain as high a yield as the membrane-assisted reactors. Once again, the FBMR stands out, starting at nearly 95% hydrogen yield and maintaining over 85% at high GHSV values. This performance is attributed to the synergy between the enhanced mass transfer in the fluidized bed and the selective permeation of hydrogen through the membrane, which together facilitate efficient hydrogen production even at higher space velocities. Overall, the FBMR demonstrates the highest methanol conversion and hydrogen yield across the range of GHSV values, making it the most efficient reactor type in this analysis. The PBMR also shows strong performance, particularly in maintaining hydrogen yield due to the membrane’s selective hydrogen removal capability. In contrast, the PBR and FBR show lower and more rapidly declining performance at higher GHSV values, with the FBR performing slightly better than the PBR due to its improved mixing and gas–solid contact. Both membrane-assisted reactors (PBMR and FBMR) significantly outperform their non-membrane counterparts, with the FBMR showing the best overall efficiency for both methanol conversion and hydrogen yield.

**Figure 8:**
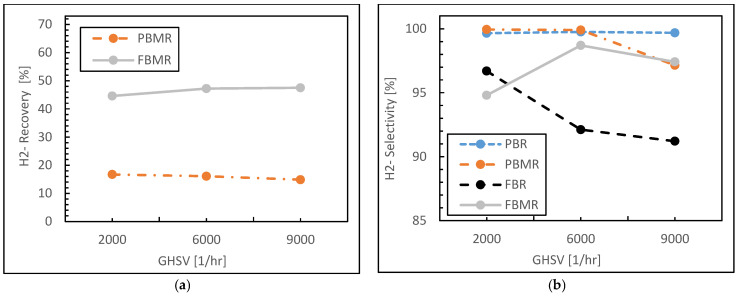
Hydrogen recovery (**a**) and Hydrogen selectivity (**b**) for FBMR in comparison with other reactors versus GHSV value; at reaction temperature 573 K, reaction pressure 1 bar, and feed molar ratio 3.

In the following, as illustrated in Figure 8a, the hydrogen recovery in the PBMR remains relatively low and stable across the range of GHSV values, starting at around 20% and slightly decreasing as GHSV increases. This suggests that, despite the use of a membrane in the PBMR, the overall hydrogen recovery efficiency is limited under the given operating conditions. The small decrease in recovery at higher GHSV values may indicate that increased flow rates reduce the contact time necessary for efficient hydrogen permeation through the membrane.

In contrast, the FBMR shows a much higher hydrogen recovery compared to the PBMR. Starting at around 40% recovery, the FBMR exhibits a slight increase as GHSV increases, maintaining recovery rates above 40% across all GHSV values. The enhanced hydrogen recovery in the FBMR can be attributed to the synergistic effects of the fluidized bed, which promotes better gas–solid mixing and turbulence, and the membrane, which selectively removes hydrogen from the reaction zone. The gradual increase in hydrogen recovery with increasing GHSV reflects the FBMR’s ability to handle higher flow rates while maintaining effective hydrogen permeation.

The hydrogen selectivity trends (see Figure 8b) are quite distinct across the four reactor configurations. The PBR and PBMR exhibit consistently high hydrogen selectivity, with values close to 100% across all GHSV values. This indicates that both reactor types are effective at maintaining high hydrogen selectivity, likely due to the lack of significant side reactions and by-product formation. The PBMR performs slightly better than the PBR, as the membrane in the PBMR helps to further ensure that hydrogen is selectively removed from the reaction zone, contributing to near-perfect selectivity. The FBR shows a significant decrease in hydrogen selectivity as GHSV increases. Starting at around 95% selectivity at low GHSV, the selectivity drops below 90% as GHSV increases. This decline suggests that the FBR is more prone to side reactions or by-product formation, particularly at higher flow rates, where increased turbulence and reduced contact time may negatively affect the reactor’s selectivity.

The FBMR exhibits initially high hydrogen selectivity, starting at around 98%, and follows a moderate decreasing trend with increasing GHSV. Despite this slight decline, the FBMR maintains selectivity above 95% at higher GHSV values. The membrane-assisted hydrogen separation in the FBMR helps preserve high selectivity, though the fluidized bed’s increased turbulence at higher flow rates may contribute to a slight reduction in selectivity as GHSV increases.

Overall, regarding GHSV evaluation, the FBMR demonstrates superior performance in hydrogen recovery compared to the PBMR, benefiting from the fluidized bed’s enhanced mass transfer and the membrane’s selective hydrogen permeation. While the PBMR’s recovery remains limited, it excels in maintaining high hydrogen selectivity comparable to the PBR. The FBR lags behind in terms of both selectivity and recovery, particularly at higher GHSV values, where its performance declines. Among all configurations, the FBMR offers a balanced and efficient approach, achieving both high hydrogen recovery and selectivity, especially under conditions of increased flow rates, making it a robust option for high-performance hydrogen production.

#### 3.1.4. Feed Molar Ratio Effect

**Figure 9:**
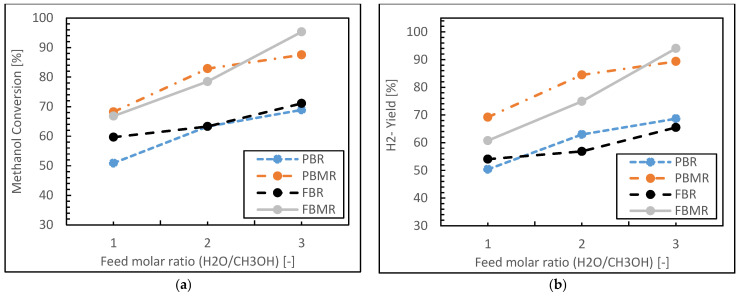
Methanol conversion (**a**) and Hydrogen yield (**b**) for FBMR in comparison with other reactors’ molar ratio (H_2_O/CH_3_OH); at reaction temperature 573 K, reaction pressure 1 bar, and GHSV 6000 h^−1^.

Figure 9 presents methanol conversion and hydrogen yield results during the MSR process for four reactor types (FBMR, PBMR, FBR, and PBR) as a function of H_2_O/CH_3_OH feed molar ratio. Both graphs demonstrate similar trends, with the reactor performance consistently following the order FBMR > PBMR > FBR > PBR for both methanol conversion and hydrogen yield across all feed ratios. The FBMR shows the highest performance, with methanol conversion (see Figure 9a) increasing from about 68% to 96% and hydrogen yield (see Figure 9b) rising from 61% to 93% as the feed ratio increases from 1 to 3. The PBMR follows closely, with conversion improving from 67% to 88% and yield from 69% to 89% over the same range. Conventional reactors show more modest improvements, with the FBR’s conversion increasing from 60% to 71% and yield from 53% to 66%, while the PBR demonstrates the lowest performance, with conversion rising from 51% to 69% and yield from 49% to 68%. All reactor types exhibit increasing trends in both methanol conversion and hydrogen yield as the H_2_O/CH_3_OH ratio increases, indicating that higher water content generally favors the reforming reaction. The MRs (FBMR and PBMR) significantly outperform conventional reactors (FBR and PBR), likely due to the continuous removal of hydrogen through the membrane, which shifts the reaction equilibrium towards products. The superior performance of fluidized bed configurations (FBMR and FBR) over their packed bed counterparts (PBMR and PBR) suggests enhanced heat and mass transfer in fluidized systems. The FBMR, combining both membrane separation and fluidization, demonstrates the highest performance across all conditions, indicating a synergistic effect that maximizes both conversion and yield. The graphs also reveal that the performance improvement, with increasing H_2_O/CH_3_OH ratio, shows signs of diminishing returns, especially for membrane reactors, suggesting an optimal feed ratio exists beyond which further increases may not be economically justifiable. These results provide valuable insights into reactor design and process optimization in MSR process, highlighting the potential of advanced reactor concepts, particularly the FBMR, for enhancing hydrogen production efficiency.

**Figure 10:**
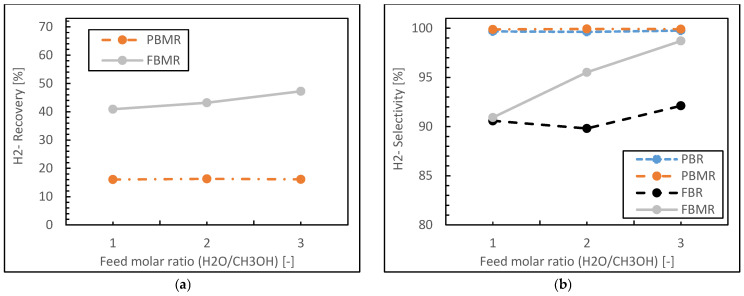
Hydrogen recovery (**a**) and Hydrogen selectivity (**b**) for FBMR in comparison with other reactors’ molar ratio (H_2_O/CH_3_OH); at reaction temperature 573 K, reaction pressure 1 bar, and GHSV 6000 h^−1^.

In the following, Figure 10 presents hydrogen recovery for MRs and hydrogen selectivity for four reactor types (PBR, PBMR, FBR, and FBMR) as a function of the H_2_O/CH_3_OH feed molar ratio during MSR.

Figure 10a shows hydrogen recovery only for the two MRs (PBMR and FBMR). The FBMR demonstrates significantly higher hydrogen recovery compared to the PBMR across all feed ratios. The FBMR’s hydrogen recovery increases from about 41% at a ratio of 1 to approximately 48% at a ratio of 3, showing a positive correlation with the feed ratio. In contrast, the PBMR exhibits a relatively constant hydrogen recovery of around 16–17% across all feed ratios, indicating little sensitivity to changes in the H_2_O/CH_3_OH ratio.

Figure 10b displays hydrogen selectivity for all four reactor types. The PBMR and PBR show nearly identical and consistently high selectivity of about 99–100% across all feed ratios. This suggests that both packed bed configurations, with and without membranes, lead to highly selective hydrogen production. The FBMR demonstrates a marked improvement in selectivity from about 91% at a ratio of 1 to 99% at a ratio of 3. The FBR shows the lowest selectivity, ranging from about 90% to 92% as the feed ratio increases.

Indeed, key points from this analysis could be presented as follows:

- Both packed bed configurations (PBMR and PBR) achieve near-perfect hydrogen selectivity across all feed ratios, regardless of membrane integration.
- The FBMR combines relatively high hydrogen recovery with increasing selectivity at higher feed ratios, making it effective for hydrogen production.
- Fluidized bed configurations (FBMR and FBR) show improved performance with increasing feed ratios, suggesting better utilization of excess water in the reaction.
- The PBMR maintains consistently high selectivity but shows lower hydrogen recovery compared to the FBMR, indicating a trade-off between these performance metrics.
- The FBR demonstrates lower selectivity compared to other reactor types, especially at lower feed ratios.

Figure 9 and Figure 10 highlight the complex interplay between reactor design, membrane integration, and operating conditions in MSR. While packed bed configurations excel in hydrogen selectivity, the FBMR offers a balance of good hydrogen recovery, methanol conversion, and improving hydrogen selectivity and yield at higher H_2_O/CH_3_OH ratios. In fact, the choice of reactor type would depend on specific process requirements, considering factors such as desired hydrogen purity, production rate, and operational complexity.

### 3.2. Components, Velocity, and Pressure Distributions

**Figure 11:**
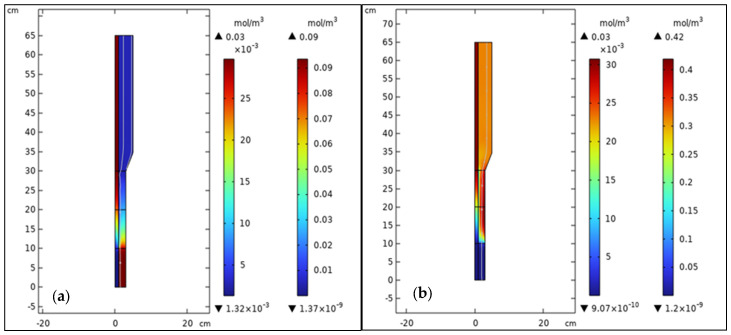
Component distribution in FBMR; (**a**) Methanol concentration; (**b**) Hydrogen concentration, at reaction pressure 5 bar, reaction temperature 573 K, GHSV of 6000 h^−1^, and feed molar ratio of 3.

Figure 11 illustrates the concentration distribution of methanol (Figure 11a) and hydrogen (Figure 11b) in the silica FBMR, obtained from a CFD simulation of the MSR process. The contour plot in Figure 11a depicts the methanol concentration distribution along the length of the FBMR. Methanol enters the reactor at the upper inlet (around 65 cm) with a high concentration, as represented by the red region in the contour plot. As methanol progresses through the reactor, its concentration decreases due to its consumption in the steam reforming reaction, which produces hydrogen, carbon dioxide, and carbon monoxide.

The contour plot in Figure 11b shows the distribution of hydrogen concentration along the reactor, which follows an inverse pattern compared to methanol. Hydrogen is produced as a result of methanol reforming, and its concentration increases along the reactor length. These results are consistent with the expected behavior in MSR, where methanol reacts with steam to produce hydrogen.

In the Supplementary File, in Figure S2, contour plots depict the velocity (Figure S2a) and pressure (Figure S2b) distributions within the FBMR. These results are crucial for understanding the fluid flow characteristics and pressure behavior in a such reactor.

### 3.3. Analysis of Bubble Regime in FBMR

**Figure 12:**
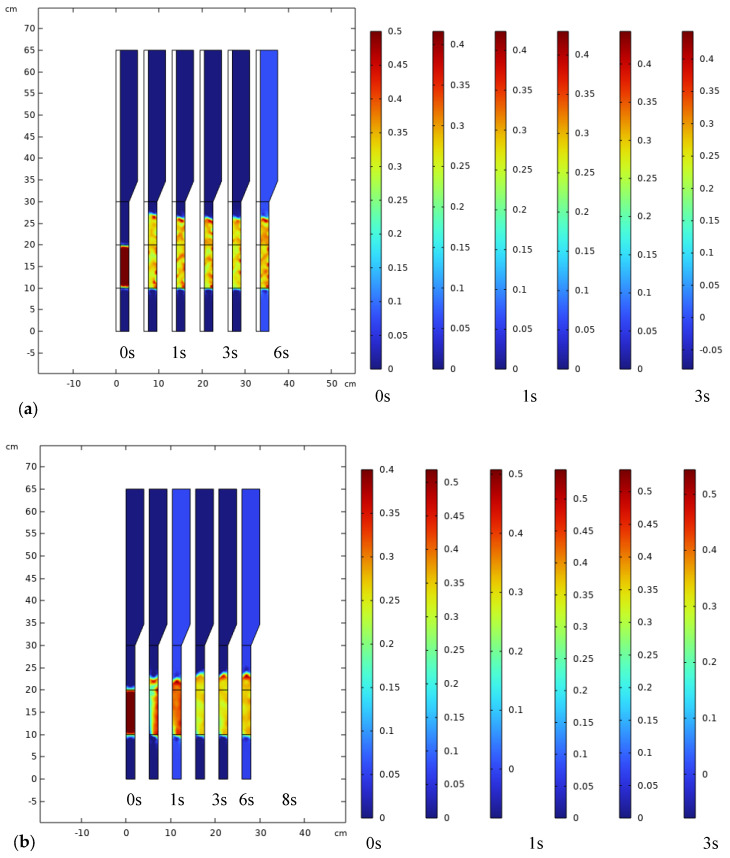
Volume fraction distribution of catalyst in FBMR (**a**) and FBR (**b**), at reaction pressure 5 bar, reaction temperature 573 K, GHSV of 6000 h^−1^, and feed molar ratio of 3.

As shown in Figure 12, the contour plot sketches the time evolution of the catalyst volume fraction in a FBMR (Figure 12a) and FBR (Figure 12b).

Initially, at 0 s, the catalyst is densely concentrated at the bottom of the reactor, indicating no fluidization. Over time (from 1 s to 6 s), the catalyst begins to move upward as fluidization progresses, with particles becoming more dispersed throughout the reactor. By 10 s, a steady-state is reached, where the catalyst is uniformly distributed across the reactor’s height. This steady fluidization ensures optimal mixing and efficient reactor performance. The comparison of different time intervals highlights the transition from a packed bed to a fully fluidized regime, which is essential for maximizing reaction efficiency in FBMR and FBR operations.

For further quantitative bubble regime analysis, to strengthen the hydrodynamic characterization of the FBMR, a quantitative analysis was conducted for various bubble rise velocities and corresponding bubble diameters. The key parameters—bubble size distribution, rise velocity, and gas–solid mass transfer coefficients—were calculated using empirical correlations based on classical fluidization theory (Davidson–Harrison and Kunii–Levenspiel models). Table 5 summarizes the results for three typical bubble rise velocity values: 0.112, 0.158 and 0.185 m/s in FBMR.

At a 0.01 m/s gas velocity, and the corresponding bubble rise velocity of 0.112 m/s, the mean bubble diameter is approximately 2.1 mm, and the calculated gas–solid mass transfer coefficient is 1.56 × 10^−3^ m/s. This condition represents a low-intensity bubbling regime with relatively limited mixing and interfacial contact. As the bubble rise velocity increases to 0.158 m/s, bubble size grows to around 4.4 mm, and the mass transfer coefficient increases to 2.91 × 10^−3^ m/s. This regime represents an optimal bubbling condition, where enhanced particle mobility and frequent bubble–emulsion phase interactions promote effective interphase mass transfer. Finally, at a higher rise velocity of 0.185 m/s—associated with bubbles of about 6.2 mm in diameter—the mass transfer coefficient reaches 3.53 × 10^−3^ m/s, indicating intensified convective mixing and turbulence. However, at such high bubbling intensities, bubble coalescence and gas channeling could potentially occur, which may reduce gas–solid contact uniformity and hydrogen yield if not carefully managed.

The observed trends suggest that moderate bubble rise velocities (around 0.158 m/s) provide a favorable trade-off between mass transfer enhancement and hydrodynamic stability. Importantly, the increase in gas–solid mass transfer coefficient with rising bubble velocity correlates well with improved hydrogen production rates observed in the CFD simulations. The fluidization regime, as inferred from these data, clearly contributes to increased reactant–catalyst contact efficiency and better distribution of reaction zones, especially in the vicinity of the membrane surface.

Overall, this analysis confirms that bubble-induced mixing and hydrodynamics play a central role in reactor performance. These findings support the use of fluidized bed configurations for membrane-assisted hydrogen production, particularly when optimized to operate within the stable bubbling regime. This improved assessment addresses the complexity of interphase transport and provides deeper insight into the dynamic behavior of catalyst particles in the FBMR.

## 4. Conclusions and Future Trends

This study presented a comprehensive CFD analysis of hydrogen production via MSR in both packed bed and fluidized bed membrane reactors. Among the configurations studied—PBR, PBMR, FBR, and FBMR—the FBMR demonstrated the highest potential for enhancing hydrogen production efficiency and methanol conversion. The superior performance of the FBMR stems from its improved gas–solid contact, enhanced mixing, and efficient in situ hydrogen separation via hydrogen-selective silica membranes. The validated CFD model provided reliable predictions of reactor behavior and highlighted the fluidized bed configuration’s capability to maintain uniform temperature distribution and strong mass transfer characteristics across the catalyst bed.

Key conclusions and optimal operating conditions from this work are summarized as follows:

Optimal Reactor Configuration and Operating Conditions:

- Reactor Type: FBMR exhibited superior hydrogen yield and uniform thermal distribution due to enhanced mixing and gas–solid interactions.
- Temperature: 250–270 °C was identified as the optimal operating window, maximizing catalyst activity while minimizing side reactions or sintering risks.
- Pressure: Slightly elevated pressures (1.5–3 bar) improved membrane permeation without compromising reaction kinetics.
- Steam-to-Methanol Ratio (S/M): A molar ratio of 1.2–1.4 was ideal to reduce energy input while avoiding coke formation.
- Membrane Features: Thin hydrogen-selective membranes with high surface area (e.g., Pd-based or silica) improved hydrogen extraction and shifted the reaction equilibrium.
- Membrane Performance Targets: Selectivity (H_2_/others) > 1000; permeability > 10^−6^ mol·m^−2^·s^−1^·Pa^−1^.
- Hydrodynamics: In the bubbling regime, bubble rise velocities of 0.15–0.25 m/s significantly enhanced gas–solid mass transfer.
- Catalyst Size and Distribution: Uniform catalyst distribution and particle diameters of 100–200 μm prevented channeling and improved reaction efficiency.

Despite these promising outcomes, it is important to acknowledge the modeling limitations inherent to this study. Firstly, the CFD simulations assumed uniform spherical catalyst particles across all reactor types. While this simplification reduces computational complexity and enables controlled comparisons, it does not fully represent real systems where polydispersity and non-spherical shapes can markedly influence fluidization behavior, bubble dynamics, and interfacial mass transfer. Future models should consider incorporating realistic particle size distributions and irregular geometries, particularly for FBMR systems, to improve the accuracy of hydrodynamic predictions—provided sufficient experimental data are available for calibration.

Additionally, the current model does not include mechanical degradation mechanisms such as catalyst attrition or membrane fatigue, which are especially relevant to long-term FBMR operation. These effects can negatively impact reactor durability and gas separation efficiency. Future work should integrate particle-based or structural mechanics simulations to quantify such degradation phenomena and evaluate their influence on sustained reactor performance.

Moreover, while the present CFD model successfully captured key reactor-level dynamics under various operating conditions, it does not include a complete energy balance or process-level energy efficiency assessment. Given the laboratory-scale dimensions of the simulated systems, a full heat and energy integration analysis may not yield practical insights at this stage. However, future studies should focus on coupling CFD results with process simulation tools such as Aspen Plus to conduct full techno-economic evaluations, including heating duties, pressure drops, fluidization energy, and potential for heat recovery—especially at industrial or pilot scales.

Finally, further research should prioritize the development of advanced, cost-effective membranes and catalysts that maintain stability and selectivity under real-world operating conditions. Integrating renewable energy sources (e.g., solar or wind) with FBMR systems, and exploring hybrid reactor architectures combining different intensification strategies, may unlock even greater efficiencies. The application of artificial intelligence and machine learning to enhance CFD optimization, process control, and predictive diagnostics presents another promising avenue to accelerate industrial deployment.

In summary, this study positions the silica-based FBMR as a high-performance, scalable, and sustainable pathway for hydrogen production, contributing meaningfully toward decarbonized energy systems.

## Figures and Tables

**Table 1:** List of parameters used in the model for silica membrane permeability for each species [14].

Gas Species, i	Q_0, i_ (mol·m^−2^·s^−1^·Pa^−1^)	E_a,i_ (kJ·mol^−1^)
H_2_	2.22 × 10^−6^	8.78
CO_2_	3.69 × 10^−8^	−1.72
CO	2.17 × 10^−7^	5.33
N_2_	8.62 × 10^−8^	3.55
H_2_O	7.11 × 10^−8^	3.49
OH_3_CH	1.68 × 10^−8^	2.52

**Table 2:** Boundary conditions used during simulations for reaction side in all configurations.

Position	Parameters	Value
Inlet (Z = 0)	F_CH3OH_ (Methanol molar flow rate)	Varied (mol/s)
Inlet (Z = 0)	F_H2O_ (Steam molar flow rate)	m. F_CH3OH_ (m = 1, 2, 3)
Inlet (Z = 0)	F_H2_ (Hydrogen molar flow rate)	1 × 10^−12^ F_CH3OH_
Inlet (Z = 0)	F_CO_ (Carbon monoxide molar flow rate)	1 × 10^−12^ F_CH3OH_
Inlet (Z = 0)	F_CO2_ (Carbon dioxide molar flow rate)	1 × 10^−12^ F_CH3OH_
Inlet (Z = 0)	F_N2_ (Nitrogen molar flow rate)	2.5 × 10^−5^ mol/s
Inlet (Z = 0)	P_inlet_	Varied (bar)
Inlet (Z = 0)	V_inlet_	Varied (m/s)

**Table 3:** Boundary conditions used during simulations for permeate side in FBMR and PBMR configurations.

Position	Parameters	Value (mol/s)
Inlet (Z = 0)	N_N2_ (Nitrogen molar flow rate)	1 × 10^−4^
Inlet (Z = 0)	N_CH3OH_	1 × 10^−12^ N_N2_
Inlet (Z = 0)	N_H2O_ (Steam molar flow rate)	1 × 10^−12^ N_N2_
Inlet (Z = 0)	N_H2_ (Hydrogen molar flow rate)	1 × 10^−12^ N_N2_
Inlet (Z = 0)	N_CO_ (Carbon monoxide molar flow rate)	1 × 10^−12^ N_N2_
Inlet (Z = 0)	N_CO2_ (Carbon dioxide molar flow rate)	1 × 10^−12^ N_N2_
Inlet (Z = 0)	P_inlet_	1 bar
Inlet (Z = 0)	V_inlet_	0.01 m/s

**Table 4:** Operating parameter values used in CFD simulations for evaluation of FBMR performance over other reactors.

Operating Parameters	Reaction Temperature	Reaction Pressure	Feed Molar Ratio	Feed Flow Rate/Catalyst Volume
T (K)	513–573	573	573	573
P (bar)	1	1–5	1	1
H_2_O/MeOH (−)	3	3	1–3	3
GHSV (h^−1^)	6000	6000	6000	2000–9000

**Table 5:** Bubble regime analysis for FBMR in different gas velocities.

Gas Velocity (m/s)	Bubble Rise Velocity (m/s)	Average Bubble Diameter (cm)	Gas–Solid Mass Transfer Coefficient (m/s)
0.01	0.112	0.21	1.56 × 10^−3^
0.02	0.158	0.44	2.91 × 10^−3^
0.04	0.185	0.62	3.53 × 10^−3^

## Data Availability

The data presented in this study are available on request from the corresponding author due to privacy.

## Supplementary Materials

The following supporting information can be downloaded at the following address: https://www.mdpi.com/article/10.3390/membranes15080248/s1. Figure S1: Publication numbers for hydrogen production via methanol reforming based on Scopus database; Figure S2: Effect of mesh numbers on calculated methanol conversion by CFD model for the four simulated reactors; FBMR, PBMR, FBR and PBR; at reaction pressure of 1 bar, reaction temperature of 573 K, GHSV of 6000 h^−1^ and H_2_O/CH_3_OH of 3; Figure S3: Velocity (a) and pressure (b) distribution in FBMR; at reaction pressure 5 bar, reaction temperature 573 K, GHSV of 6000 h^−1^, and feed molar ratio of 3. Table S1: Kinetic and termodynmaic data used in CFD simulation; Table S2: List of parameter values used in the simulation; Table S3: Results of mesh independency for four reactor schemes.

## Abbreviations

Acronym	
FBMR	Fluidized bed membrane reactor
FBR	Fluidized bed reactor
GHSV	Gas hourly space velocity
MR	Membrane reactor
MD	Methanol decomposition
MSR	Methanol steam reforming
PBMR	Packed bed membrane reactor
PBR	Packed bed reactor
SR	Steam reforming
WGS	Water gas shift
Nomenclature	
A	Cross area of retentate side, m^2^
$C_{i}^{T}$	Total surface concentration of site i, mol m^−2^
CH_3_OH_in_	Methanol molar flow rate fed to the reactor, mol s^−1^
CH_3_OH_out_	Methanol molar flow rate going out from the reactor, mol s^−1^
D	Membrane diameter, m
d_p_	Catalyst diameter, m
E_a_	Apparent activation energy for membrane, J mol^−1^
E_a,i_	Transport activation energy for species i in the silica membrane, J mol^−1^
E_i_	Activation energy for rate constant of reaction i, kJ mol^−1^
F_CH3OH_	Molar methanol inlet flow rate, mol s^−1^
ΔH_i_	Heat of adsorption for surface species iii or heat of reaction for formation of surface Species i, kJ mol^−1^
J_i_	Flux of component i, mol m^2^ s^−1^
k_j_	Rate constant for reaction j (units are specific to the form of the rate expression)
$k_{i}^{\infty }$	Pre-exponential term in Arrhenius expression; this is the intercept of the Arrhenius Equation as T→∞
$k_{j}^{eq}$	Equilibrium constant of reaction j
$k_{i}^{*}$	Equilibrium constant or adsorption coefficient for surface species i
m	Feed molar ratio coefficient
N_i_	Molar flow rate, mol m^2^ s^−1^
P	Total pressure, bar
p_i_	Partial pressure of component i, bar
${Pe}_{i}^{*}$	Permeance coefficient for silica membranes of species i, mol s^−1^ cm^−2^ atm^−0.5^
Pe	Permeability coefficient, mol s^−1^ cm^−1^ atm^−0.5^
$P_{H_{2},lumen}$	Hydrogen partial pressure in retentate side, bar
$P_{H_{2},sheell}$	Hydrogen partial pressure in permeate side, bar
r_i_	Rate of reaction i, mol s^−1^ m^−2^, or rate of formation of species i, mol s^−1^g_catalyst_^−1^
R	Gas constant, kPa m^3^ mol^−1^ K^−1^
ΔS_i_	Entropy of adsorption for species i, J mol^−1^ K^−1^
S_A_	Surface area of fresh catalyst, m^2^ g^−1^
T	Temperature, K
V	Volume of catalyst or reactor, m^3^
U	Gas velocity, m s^−1^
W	Mass of catalyst, g
z	Axial coordinate
Greek letter	
ρ_g_	Density of gas, kg m^−3^
Ρ_b_	Density of the catalyst bed, kg m^−3^
μ	Gas viscosity, kg m^−1^ s^−1^
δ	Membrane thickness, m
ε	Porosity of the catalyst
ν_ij_	Stoichiometric coefficient
