# Supplementary material for: Performance Analysis of Silica Fluidized Bed Membrane Reactor for Hydrogen Production as a Green Process Using CFD Modelling
Source: Membranes (Basel). 2025 Aug 18;15(8):248. doi: 10.3390/membranes15080248 (PMC12388487; doi:10.3390/membranes15080248)
Supplement: Supplementary file 1 [file membranes-15-00248-s001.zip › membranes-3740365-supplementary.pdf]

<u>**Supplementary Data**</u>

Performance Analysis of Silica Fluidized Bed Membrane Reactor for Hydrogen Production as a Green Process Using CFD Modelling

Maryam Barmaki [1], Elham Jalilnejad [1], Kamran Ghasemzadeh [1,2,*] and Adolfo Iulianelli [3,*]

[1] Chemical Engineering Department, Urmia University of Technology, Urmia 57155-419, Iran; mmb.barmaki1378@gmail.com (M.B.); e.jalilnejad@uut.ac.ir (E.J.)
[2] Institute for Materials and Processes, School of Engineering, University of Edinburgh, Edinburgh EH8 9YL, UK
[3] Institute on Membrane Technology of the National Research Council (CNR-ITM), via P. Bucci 17C,
87036 Rende, Cosenza Italy
* Correspondence: kghasemz@ed.ac.uk (K.G.); a.iulianelli@itm.cnr.it (A.I.)

1. Introduction

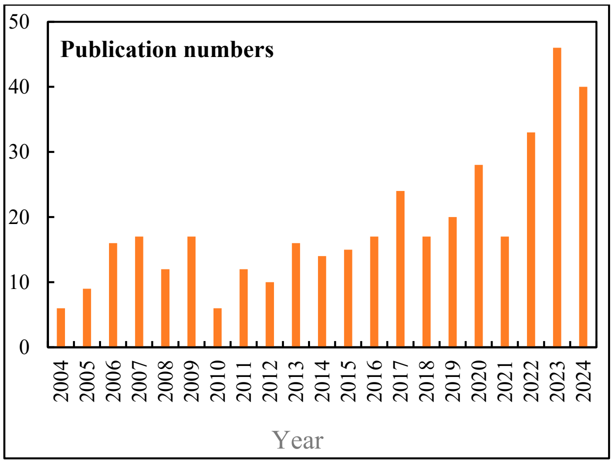

Figure S1. Publication numbers for hydrogen production via methanol reforming based on Scopus database

2. Methodology

2.2. CFD Modelling approach

2.2.1. Governing equations

- Turbulence Modelling Approach

To account for the turbulence effects associated with gas–solid interactions in the FBMR and FBR cases, the Renormalization Group (RNG) k–ε turbulence model was employed. This model is particularly suited for capturing the complex, anisotropic turbulent structures prevalent in bubbling and turbulent fluidization regimes. Unlike the standard k–ε model, the RNG k–ε formulation includes an additional term in the ε-equation to account for the interaction between turbulence dissipation and mean shear strain, offering improved accuracy in swirling and recirculating flows. The governing transport equations for turbulent kinetic energy (k) and its dissipation rate (ε) are expressed as:

$$\frac{\partial(\rho k)}{\partial t}+\frac{\partial(\rho k u_i)}{\partial x_i}=\frac{\partial}{\partial x_j}\left(\left(\mu+\frac{\mu_t}{\sigma_k}\right)\frac{\partial k}{\partial x_j}\right)+G_k-\rho\varepsilon$$

$$\frac{\partial(\rho\epsilon)}{\partial t}+\frac{\partial(\rho\varepsilon u_i)}{\partial x_i}=\frac{\partial}{\partial x_j}\left(\left(\mu+\frac{\mu_t}{\sigma_\varepsilon}\right)\frac{\partial k}{\partial x_j}\right)+G_{1\varepsilon}\frac{\varepsilon}{k}G_k-C_{2\varepsilon}\rho\frac{\varepsilon^2}{k}-R$$

ρ: Fluid density (kg/m^3)

k: Turbulent kinetic energy (m^2/s^2)

u_i: Velocity component in the i-th direction (m/s)

t: Time (s)

x_i, x_j : Spatial coordinates in Cartesian directions (m)

μ: Molecular (dynamic) viscosity (Pa·s)

μ_t : Turbulent eddy viscosity (Pa·s)

σ_k: : Turbulent Prandtl number for

G_k: Production of turbulent kinetic energy due to mean velocity gradients (W/m^3)

ε: Rate of dissipation of turbulent kinetic energy (m^2/s^3)

σ_ε : Turbulent Prandtl number for

$C_{1\varepsilon}$: Model constant (~1.42 in standard RNG)

$C_{2\varepsilon}$: Model constant (~1.68 in RNG model)

R: Extra term specific to RNG model accounting for strain-rate effects (W/m^3)

where G_k represents the generation of turbulence kinetic energy due to mean velocity gradients, and μ_t is the turbulent viscosity defined as:

$$\mu_t = \rho C_\mu \frac{k^2}{\varepsilon}$$

The RNG model introduces a correction term R in the ε-equation and uses different model constants ($C\mu$=0.0845) derived from renormalization group theory. This model has been successfully applied in prior studies of fluidized bed reactors and is available in COMSOL Multiphysics, where it was used in combination with the Eulerian–Eulerian multiphase formulation to resolve turbulence–particle interactions accurately. The selected approach ensures robust prediction of momentum exchange, gas dispersion, and enhanced interphase mass transfer, all of which are critical to accurately simulating the FBMR dynamics under varying inlet velocities.

- Reaction rate constant equations:

$$k_{SR} = k_{SR}^{\infty} \exp\left(\frac{-E_{SR}}{RT}\right)$$

$$k_{MD} = k_{MD}^{\infty} \exp\left(\frac{-E_{MD}}{RT}\right)$$

$$k_{WGS}^{*} = E_{WGS}^{\infty^*} \exp\left(\frac{-E_{WGS}^{*}}{RT}\right)$$

- Adsorption constant equations:

$$K_i^{*} = \exp\left(\frac{\Delta S_i}{R} - \frac{\Delta H_i}{RT}\right)$$

- Rate of formation or consummation of species:

$$r_{CO_2} = (r_{SR} + r_{WGS})S_A$$

$$r_{CO} = (r_{MD} - r_{WGS})S_A$$

$$r_{H_2} = (3r_{SR} + 2r_{MD} + r_{WGS})S_A$$

$$r_{CH_3OH} = (-r_{SR} - r_{MD})S_A$$

$$r_{H_2O} = (-r_{SR} - r_{WGS})S_A$$

- Equilibrium information:

$$K_{WGS}^{eq} = \exp\left(\frac{-E_{WGS}}{RT} + \frac{\Delta S_{WCS}}{R}\right)$$

$$K_{MD}^{eq} = \exp\left(\frac{-E_{MD}}{RT} + \frac{\Delta S_{MD}}{R}\right)$$

$$K_{SR}^{eq} = \exp\left(\frac{-E_{SR}}{RT} + \frac{\Delta S_{SR}}{R}\right)$$

$$E_{SR} = 48950\ \mathrm{J/mol}$$

$$E_{WGS} = -41180\ \mathrm{J/mol}$$

$$E_{MD} = 90130\ \mathrm{J/mol}$$

$$K_{SR}^{eq} = 42.4\mathrm{atm}^2$$

$$K_{WGS}^{eq} = 86.45\mathrm{atm}^0$$

$$K_{MD}^{eq} = 219.9\mathrm{atm}^2$$

Table S1. Kinetic and termodynmaic data used in CFD simulation

**Kinetcis data**			**Adsoption data**		
$k_j = k_j^{\infty} \exp\left(\frac{-E_j}{RT}\right)$			$\mathbf{K_i^{*} = \exp\left(\frac{\Delta S_i}{R} - \frac{\Delta H_i}{RT}\right)}$		
k_j	$k_j^{\infty}[m^2 s^{-1} mol^{-1}]$	$E_j[kj/mol]$	K_i^{∞}	$\Delta S_i[j.\,mol^{-1}.\,k^{-1}]$	$\mathbf{\Delta H_i[kj/mol]}$

k_{SR}	7.4×10^{14}	102.8	$k^*_{CH_3O^{(1)}}$	-41.8	-20
k_{MD}	3.8×10^{20}	170.0	$k^*_{CH_3O^{(2)}}$	30	-20
k_{WGS}	5.9×10^{13}	87.6	$k^*_{HCOO^{(1)}}$	179.2	100
			$k^*_{OH^{(1)}}$	-44.5	-20.0
			$k^*_{OH^{(2)}}$	30	-20.0
			$k^*_{H(1a)}$	-100.8	-50.0

Table S2. List of parameter values used in the simulation.

Parameter	Values
Silica membrane thickness (m)	1×10^{-7}
Silica membrane outer diameter (m)	1×10^{-2}
Silica membrane length (m)	50×10^{-2}
Catalyst particle size (m)	5×10^{-4}
Catalyst particle surface area (m^2/g)	50
Catalyst bed density (kg/m^3)	1300
Catalyst bed porosity	0.6
Mass of catalyst (g)	6
C^T_{s1} (mol/m^2)	7.5×10^{-6}
C^T_{s2} (mol/m^2)	7.5×10^{-7}
C^T_{s1a} (mol/m^2)	2×10^{-6}
C^T_{s2a} (mol/m^2)	1.5×10^{-6}
Pe_0 (mol/s.cm.$atm^{0.5}$)	2.69×10^{-7}
E_a(J/mol)	10580
R(m^3/atm mol K)	8.314

2.3. Solving method and mesh independency

Table S3. Results of mesh independency for four reactor schemes

Reactor Type	Mesh size	Methanol Conversion (%)	Pressure drop (Pa)
**Packed Bed Reactor**	6212	63.658	1659
	8792	67.361	1581
	**15477**	68.843	1492
	21839	68.907	1491
**Packed Bed Membrane Reactor**	8531	82.264	1647
	12213	86.298	1565
	**16077**	87.497	1491
	22111	87.639	1489
**Fluidized Bed Reactor**	7395	66.157	1329
	11335	70.547	1253
	**17021**	71.072	1191
	25356	71.224	1191
**Fluidized Bed Membrane Reactor**	11055	90.525	1313
	18620	93.917	1243
	**27202**	95.277	1190
	33762	95.537	1189

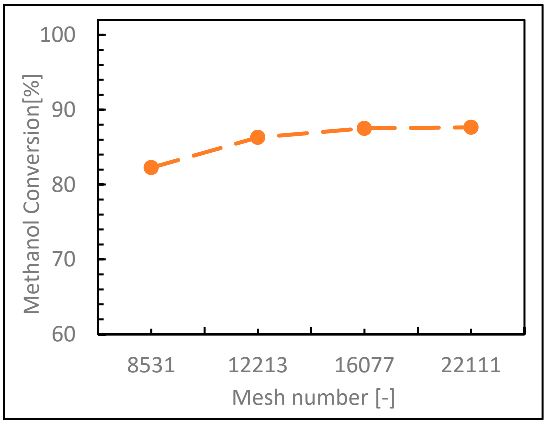

Packed Bed Membrane Reactor

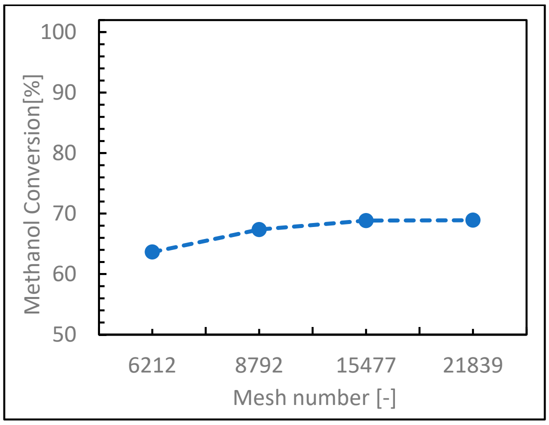

Packed Bed Reactor

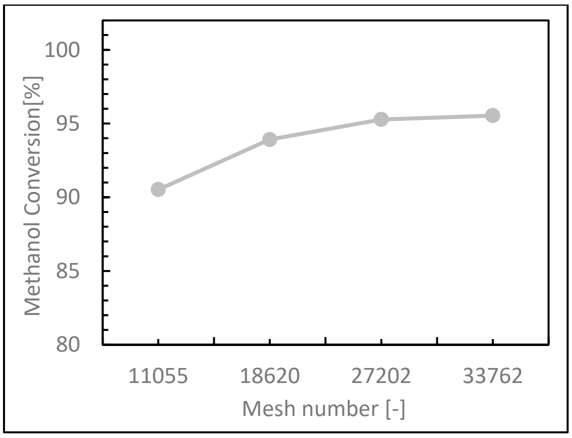

Fluidized Bed Membrane Reactor

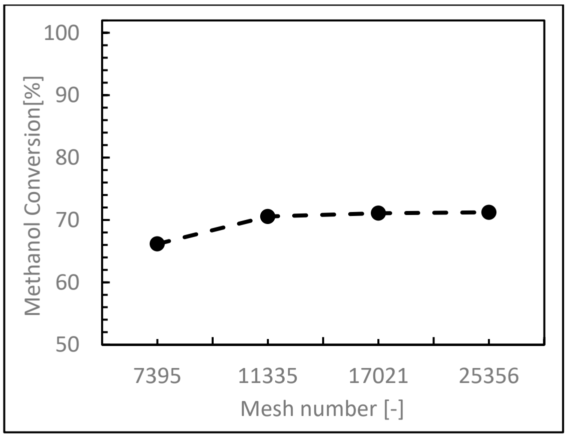

Fluidized Bed Reactor

Figure S2. Effect of mesh numbers on calculated methanol conversion by CFD model for the four simulated reactors; FBMR, PBMR, FBR and PBR; at reaction pressure of 1 bar, reaction temperature of 573 K, GHSV of 6000 h^{-1} and H_2O/CH_3OH of 3.

3. Result and discussion

3.2. Components, velocity, and pressure distributions

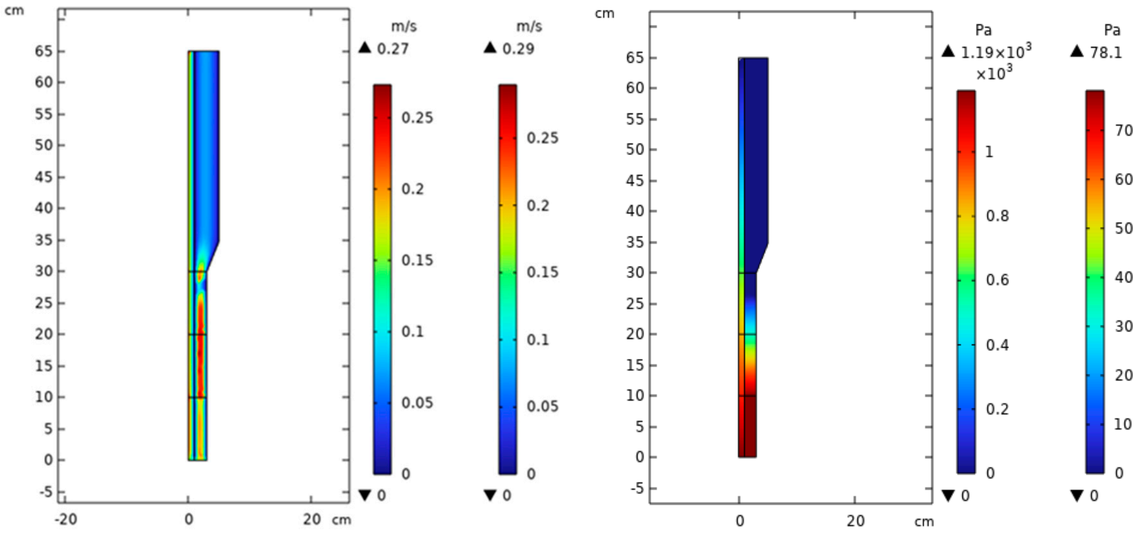

Figure S3. Velocity (a) and pressure (b) distribution in FBMR; at reaction pressure 5 bar, reaction temperature 573 K, GHSV of 6000 h^{-1}, and feed molar ratio of 3.
